# Quantification of gait in mitochondrial m.3243A > G patients: a validation study

**DOI:** 10.1186/s13023-017-0644-y

**Published:** 2017-05-15

**Authors:** Rob Ramakers, Saskia Koene, Jan T Groothuis, Paul de Laat, Mirian CH Janssen, Jan Smeitink

**Affiliations:** 1 Radboud Center for Mitochondrial Medicine (RCMM) at the Department of Pediatrics, Radboud University Medical Center Nijmegen, Geert Grooteplein Zuid 10, PO BOX 9101, 6500 HB Nijmegen, The Netherlands; 2 Radboud Center for Mitochondrial Medicine (RCMM) at the Department of Internal Medicine, Radboud University Medical Center Nijmegen, Geert Grooteplein Zuid 10, PO BOX 9101, 6500 HB Nijmegen, The Netherlands; 3 Department of Rehabilitation, Donders Institute for Brain, Cognition and Behaviour, Radboud University Medical Center, PO BOX 9101, 6500 HB Nijmegen, The Netherlands

**Keywords:** Gait, Mitochondrial disease, m.3243A > G, MELAS, Trial

## Abstract

**Background:**

More than half of the patients harbouring the m.3243A > G mutation were found to have trouble maintaining balance when walking in a recent study by our group. Others demonstrated that these patients had an abnormal gait pattern, as quantified by gait analysis. Gait analysis is an emerging method to quantify subtle changes in walking pattern, also during therapeutic interventions. Therefore, we aimed to test the reliability and reproducibility of gait analysis and select the most suitable protocol for this group of patients using a GAITRite electronic walkway. Four different protocols were tested: normal walking, dual task, post exercise and after a ten minutes of rest.

**Results:**

In total 36 patients with the m.3243A > G mutation and 50 healthy controls were enrolled in this study. Overall high intra class correlation coefficients were found in all experimental conditions for both patients and healthy controls indicating good reproducibility. Marked differences in gait between patients and controls were observed and were in line with the only available exploratory study performed. There was a good correlation between both the overall NMDAS score, NMDAS subscale scores, both markers for disease severity, and specific gait parameters.

**Conclusions:**

The observed reliability of the test makes GAITRite a suitable instrument for intervention studies in patients with mitochondrial disease.

## Background

Over 1150 genes encoding mitochondrial proteins have been identified with the use of mass spectrometry of mitochondria [1, 2]. Mutations in genes encoding these mitochondrial proteins, like those involved in oxidative phosphorylation, can cause mitochondrial disease. Abnormal mitochondrial functioning has a major impact, especially prone being high energy dependent tissues and organs like skeletal muscles and the brain [3]. With an estimated total prevalence of roughly 1 in 4300 adults, mitochondrial disease is one of the most common inherited neuromuscular conditions of metabolism [4]. Among these mutations the m.3243A > G mutation is one of the most prevalent [4].

Up until this date there is no definite clinical beneficial treatment for mitochondrial patients [4]. A recent Cochrane review investigated the effect of various treatment strategies in patients with mitochondrial disease and concluded that treatment efficacy was difficult to assess as a result of large variations in disease phenotype and different study endpoints. It was suggested that future studies should therefore focus on as much as possible homogenous signs and symptoms present in these patients.

Recently, we investigated the different clinical symptoms in a group of mitochondrial patients harbouring the m.3243A > G mutation. This genotype is associated with a plethora of symptoms including e.g. diabetes mellitus, myopathy, deafness, cardiomyopathy and MELAS (mitochondrial encephalomyopathy, lactic acidosis and stroke-like episodes) syndrome [5]. The results of our study indicated that about 54% of the subjects in this study had a decreased exercise tolerance and 51% had trouble maintaining balance while walking [6]. Recently, Galna et al. quantified the gait of these patients and showed that patients with the m.3243A > G mutation have an abnormal walking pattern compared to healthy controls [7].

The GAITRite is used in numerous other diseases and conditions including Parkinson’s disease, stroke, cerebellar ataxia and aging [8–13]. It is an emerging method to quantify subtle changes in walking pattern [8–11] also during therapeutic interventions [12, 13]. However, there are no previous studies that focused on the reliability of these measurements in mitochondrial patients.

This study aims to optimize the protocol to quantify gait patterns in mitochondrial patients with the m.3243A > G mutation.

## Methods

This prospective study aims to select the most reliable and valid gait-quantification protocol by testing four different walking conditions in mitochondrial patients. We examined the reliability of the gait pattern analysis of a clinically heterogeneous group of mitochondrial patients with the m.3243A > G mutation as well as healthy controls during either normal walking, during dual tasks and after exercise. Secondly, we compared the gait patterns of the mitochondrial patients with healthy controls to gain more insight in the difference between both groups. We hypothesized that gait assessment in patients is reliable and we expected the best reliability in the normal walking condition. Finally, we hypothesized that the gait characteristics of the patients differ from healthy controls.

We quantified gait characteristics of 39 patients above the age of 18 harbouring the m.3243A > G mutation in the tRNA^leu (UUR)^ and 50 healthy adult controls. Exclusion criteria for this study were: any other disease causing abnormal gait pattern (e.g. orthopaedic, other neurological or neuromuscular diseases) and the inability to complete a 3-min walking test. The heteroplasmy levels in patients were evaluated in blood, urine and buccal smears and/or skeletal muscle biopsy samples. Participants were not given or encouraged to take any additional medication or supplements throughout this study. However, we did not keep track of non-prescribed medication or supplement intake. The ethics committee of Arnhem-Nijmegen region, The Netherlands, approved this study. Written informed consent was obtained from all participants.

### Measurements

Upon arrival height and weight were measured and patients were screened on disease status using the Newcastle Mitochondrial Disease Adult Scale (NMDAS) [14]. The NMDAS constitutes a validated method to monitor the clinical expression of mitochondrial disease and to follow-up the course of disease in time. The NMDAS consists of the following four sections. 1) Current function, gives insight into the general functioning of patients in past four weeks. 2) System-specific involvement uses a clinical history supplemented by specific information to gain insight in the functioning of individual organ-systems. 3) Current clinical assessment, a general and neurological clinical examination, gives insight in the current functional status of the patient. 4) Quality of life, we used a Dutch translation of the Short Form-36 (SF-36) quality of life test.

**Fig. 1:**
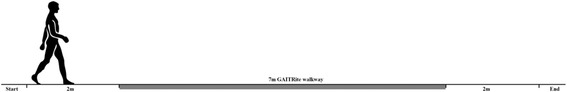
Schematic representation of the GAITRite walkway setup. Subjects started 2 m in front of and ended 2 m behind the actual GAITRite walkway to ensure they would have a constant walking speed

Leg length was measured as true leg length, from the Anterior Superior Iliac Spine (ASIS) towards the medial malleolus on both sides. The force of the quadriceps muscle (m. quadriceps) was assessed using a Micro-Fet hand held dynamometer (Hoggan health industries, Salt Lake City, USA). The Berg Balance Scale (BBS) was performed to measure dynamic and static balance [15]. A portable GAITRite electronic walkway system was used to quantify gait patterns (Platinum model GAITRite, software version 4.7, CIR systems, USA). The system was set up in a lab setting and consisted of a 7 m long walkway with 2 m free walking space at both ends for acceleration (Fig. 1).

### Gait assessment

**Fig. 2:**
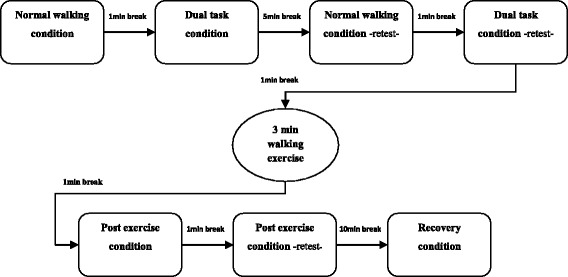
Gait assessment flowchart. All subjects completed the same rotation of trial conditions and were given the same resting perioid. Each condition consisted of three walks across the GAITRite walkway

Participants were instructed to walk across the GAITRite-mat at their self-selected pace under different conditions (Fig. 2). Each measurement condition existed of three walks (trials) across the mat without breaks in between. Participants started with the normal walking condition, in which they were given no further tasks other than walking at their self-selected pace. After a one-minute break, the participant was given the same instruction as with the normal walking condition now with one additional task (e.g. subtracting 7 from 100 and beyond). After a five-minute break, both the normal walking and the dual task conditions were repeated with a 1 min break in between. Subsequently, participants performed a 3-min walking distance test (3MWT), to induce tiredness. After a 1-min break, participants were instructed to walk over the mat without any other tasks (post exercise condition). This measurement was repeated twice after a 1-min break and a 10 min break, respectively. The last condition (after the 10-min break) is thought of as a recovery condition (i.e. does the participant recover from the walking test and how does this affect the walking pattern). The order of the protocol was fixed to prevent influences of the exercise-induced exhaustion with the normal walking and dual task conditions.

### Data processing

**Fig. 3:**
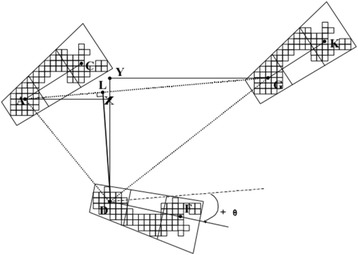
Schematic representation of the calculations done on spatial gait parameters by the GAITRite software. Stride length left foot (−AG); Step length right foot (−AX); Step length left foot (−GY); Base of support/Base width of the right foot (−LD)

We’ve focused our gait analysis based on the model introduced by Lord et al. in 2013 [13]. This model consists of five domains for assessing gait including pace (step length and step velocity), rhythm (step time), variability (step length and step time variability), asymmetry (step time asymmetry) and postural stability (step width, step width variability and step length asymmetry) [13]. These domains are also reflected in the clinical symptoms of patients with the m.3243A > G mutation (e.g. losing balance, ataxic gait pattern). Mean velocity, step length, step time and step width were calculated by the GAITRite software automatically for each trial and for each condition (Fig. 3). Variability was calculated as the root of the mean variance of the left and right foot. Standard deviations for step length, step time and step width were therefore calculated per trial based on the individual steps in the trial. Step time and step length asymmetry was defined as the absolute difference between both feet.

### Statistical analysis

All data are presented as mean ± standard deviation unless otherwise specified. To test the reliability of the gait parameters measured with the GAITRite, Intraclass Correlation Coefficients (ICCs) were calculated for each study group, variable and condition. Since this is an exploratory study, statistical significance has been defined as *p* < 0 · 05. ICCs above 0 · 8 were defined as ‘good’ and above 0 · 9 ‘perfect’. Furthermore, we tested differences between both study groups using the non-parametrical Mann–Whitney *U* test in non-Gaussian distribution, statistical significance has been defined as *p* < 0 · 05 (two-tailed). Correlation analysis was carried out between the NMDAS and the different gait characteristics for the normal walking condition only. Subscales of the NMDAS scoring for exercise intolerance, gait instability, myopathy and cerebral ataxia were correlated with gait characteristics of the normal walking condition. Additionally, we tested the correlation between the heteroplasmy levels in blood and urine with the gait characteristics in the normal walking condition.

## Results

In total 36 out of 39 patients with the m.3243A > G mutation and 50 healthy controls were included in the final analysis of this study. Three patients had to be excluded from participation, of which two patients had orthopaedic condition that caused an abnormal gait pattern and one patient could not perform the tasks that were given for the specific walking conditions (i.e. 3-min walking test and calculating). The physical characteristics of the patients and controls and the disease specific characteristics of the patients are presented in Table 1. Patients were significantly older and shorter compared to the adult control group but did not differ significant in weight (Table 1). Furthermore, patients scored lower on the BBS, walked a shorter distance during the 3MWT and had a lower maximal force of the m. quadriceps (Table 1).

**Table 1:** Study population characteristics

	Patients (*N* = 36)	Controls (*N* = 50)
Gender (N female)	26	30
Age (years)	48 · 5 ± 11 · 2	38 · 6 ± 14 · 9*
Height (cm)	169 · 3 ± 9 · 7	175 · 2 ± 9 · 6*
Weight (kg)	69 · 1 ± 11 · 2	74 · 4 ± 13 · 0
Heteroplasmy in blood (%)	18 · 5 ± 11 · 1	-
Heteroplasmy in urine (%)	53 · 1 ± 21 · 3	-
NMDAS (section 1–3)	17 · 2 ± 11 · 6	-
BBS	53 · 6 ± 3 · 8	56 · 0 ± 0 · 0†
3MWD (m)	265 · 3 ± 44 · 5	322 · 6 ± 31 · 6†
F_max_ m. quadriceps (N)	250 · 7 ± 89 · 3	340 · 0 ± 72 · 4†

*NMDAS* Newcastle Mitochondrial Disease Adult Scale, *BBS* Berg Balance Scale, *3MWD* Distance on the 3 min walking test, *F*_*max*_ *m. quadriceps*: maximal force of both legs measured in the m. quadriceps

significantly different from patients

*p < 0 · 05 †*p* < 0 · 001

### Reliability

ICCs of the gait parameters for the different conditions are presented in Table 2. All ICCs in both the patient and control group were significant with a *p*-value <0 · 001. The best ICCs were found in the post exercise condition for the patient group and in the after resting condition for the control group. Overall high ICCs were found in all conditions, except for the step time variable in the normal and dual task condition scored below 0 · 70 in the patient group (Table 2).

**Table 2:** Intra class correlations coefficients (ICCs) of the gait parameters for each walking condition

	Variable	Normal walking	Dual task	Post exercise	After rest
Patients	Velocity (cm/s)	*0 · 89*	*0 · 88*	**0 · 92**	**0 · 92**
	Step length L (cm)	**0 · 92**	*0 · 83*	**0 · 94**	**0 · 91**
	Step length R (cm)	**0 · 90**	*0 · 87*	**0 · 94**	**0 · 93**
	Step time L (s)	0 · 64	0 · 72	**0 · 91**	**0 · 90**
	Step time L (s)	*0 · 88*	0 · 67	**0 · 90**	**0 · 91**
	Step time L (s)	0 · 77	*0 · 86*	*0 · 83*	0 · 74
	Step time L (s)	*0 · 80*	*0 · 85*	*0 · 84*	0 · 76
Controls	Step time L (s)	*0 · 87*	**0 · 92**	*0 · 89*	**0 · 94**
	Step time L (s)	**0 · 90**	*0 · 88*	**0 · 92**	**0 · 93**
	Step time L (s)	**0 · 90**	*0 · 89*	**0 · 90**	**0 · 93**
	Step time L (s)	**0 · 90**	*0 · 85*	*0 · 89*	**0 · 94**
	Step time L (s)	*0 · 89*	*0 · 89*	**0 · 90**	**0 · 94**
	Step time L (s)	0 · 79	*0 · 80*	0 · 70	0 · 76
	Step time L (s)	*0 · 80*	0 · 78	0 · 70	0 · 74

*R* right foot, *L* Left foot

ICCs in *italic* are good (above 0 · 80) and ICCs in **bold** are perfect (above 0 · 90), all ICCs were significant with a *p*-value <0 · 001

### Gait characteristics

Comparisons of the gait characteristics per group for each condition are shown in Table 3. In all of the conditions we found a significantly lower gait velocity and shorter step length in the patient group. Furthermore, patients had higher step width variability in all but the post exercise condition whilst having a similar mean step width. There were no significant differences in step time and step length asymmetry in all conditions. Results also show that both controls and patients tend to walk slowest during dual task conditions and the fastest during the post exercise condition.

**Table 3:** Gait characteristics per walking condition

Condition	Variable	Patients (*N* = 36)	Controls (*N* = 50)	Difference between Patients and Controls (*p*-value)
Normal walking	Velocity (m/s)	1 · 11 ± 0 · 19	1 · 32 ± 0 · 16	**<0 · 001**
	Step length (m)	0 · 59 ± 0 · 08	0 · 69 ± 0 · 06	**<0 · 001**
	Step time (s)	0 · 54 ± 0 · 05	0 · 53 ± 0 · 05	0 · 135
	Step width (m)	0 · 10 ± 0 · 03	0 · 09 ± 0 · 03	0 · 406
	Step length variability (mm)	29 · 1 ± 14 · 4	25 · 2 ± 7 · 8	0 · 241
	Step time variability (ms)	28 · 6 ± 33 · 2	19 · 2 ± 7 · 1	*0 · 013*
	Step width variability (mm)	25 · 4 ± 7 · 4	20 · 1 ± 5 · 7	**<0 · 001**
	Step Length Asymmetry (mm)	16 · 3 ± 14 · 3	15 · 4 ± 11 · 4	0 · 986
	Step Time Asymmetry (ms)	8 · 6 ± 8 · 7	8 · 0 ± 8 · 3	0 · 759
Dual task	Velocity (m/s)	0 · 93 ± 0 · 21	1 · 16 ± 0 · 23	**<0 · 001**
	Step length (m)	0 · 57 ± 0 · 08	0 · 65 ± 0 · 07	**<0** · **001**
	Step time (s)	0 · 64 ± 0 · 16	0 · 58 ± 0 · 12	*0* · *036*
	Step width (m)	0 · 10 ± 0 · 04	0 · 10 ± 0 · 03	0 · 703
	Step length variability (mm)	40 · 6 ± 24 · 4	29 · 3 ± 9 · 6	*0* · *002*
	Step time variability (ms)	79 · 4 ± 106 · 2	37 · 8 ± 59 · 6	**<0** · **001**
	Step width variability (mm)	27 · 2 ± 9 · 0	23.0 ± 6 · 7	*0* · *019*
	Step Length Asymmetry (mm)	19 · 2 ± 21 · 9	16.7 ± 14 · 4	0 · 875
	Step Time Asymmetry (ms)	18 · 6 ± 21 · 8	15.4 ± 18 · 2	0 · 578
Post exercise	Velocity (m/s)	1 · 21 ± 0 · 18	1 · 45 ± 0 · 15	**<0** · **001**
	Step length (m)	0 · 62 ± 0 · 08	0 · 72 ± 0 · 06	**<0** · **001**
	Step time (s)	0 · 52 ± 0 · 05	0 · 50 ± 0 · 04	0 · 070
	Step width (m)	0 · 10 ± 0 · 03	0 · 09 ± 0 · 02	0 · 391
	Step length variability (mm)	27 · 7 ± 10 · 2	24 · 4 ± 7 · 8	0 · 109
	Step time variability (ms)	20 · 4 ± 10 · 7	16 · 8 ± 5 · 3	0 · 168
	Step width variability (mm)	23 · 6 ± 7 · 2	21 · 0 ± 5 · 6	0 · 089
	Step Length Asymmetry (mm)	17 · 8 ± 14 · 0	15 · 9 ± 11 · 3	0 · 796
	Step Time Asymmetry (ms)	10 · 3 ± 11 · 8	8 · 2 ± 7 · 2	0 · 666
After rest	Velocity (m/s)	1 · 18 ± 0 · 18	1 · 42 ± 0 · 16	**<0** · **001**
	Step length (m)	0 · 60 ± 0 · 08	0 · 71 ± 0 · 06	**<0** · **001**
	Step time (s)	0 · 52 ± 0 · 05	0 · 50 ± 0 · 04	0 · 102
	Step width (m)	0 · 10 ± 0 · 03	0 · 09 ± 0 · 02	0 · 439
	Step length variability (mm)	26 · 4 ± 12 · 1	20 · 7 ± 7 · 6	*0* · *023*
	Step time variability (ms)	19 · 1 ± 9 · 6	15 · 2 ± 4 · 4	0 · 054
	Step width variability (mm)	24 · 0 ± 9 · 1	19 · 5 ± 5 · 6	*0* · *027*
	Step Length Asymmetry (mm)	15 · 8 ± 14 · 6	16 · 4 ± 10 · 1	0 · 365
	Step Time Asymmetry (ms)	11 · 2 ± 12 · 0	8 · 0 ± 7 · 3	0 · 409

Differences in gait characteristics between patients with the m.3243A > G mutation and healthy controls, significant *p*-values below *p* = 0 · 05 are shown in *italic*, *p*-values below *p* = 0 · 001 are shown in **bold**

### Correlation analysis

Higher scores in the NMDAS questionnaire, reflecting a more severe disease status, was inversely correlated with gait velocity and step length, and positively correlated with step length variability and step length variability in a normal walking condition (Table 4). However, the NMDAS did not correlate with step width, step width variability, step length asymmetry and both step time and, step time asymmetry. All four of the examined NMDAS subscales were negatively correlated with both step velocity and step length. Furthermore, the gait instability and cerebellar ataxia scales were positively correlated with step length variability, step time variability and, step width variability. Step length asymmetry was only positively correlated with the exercise intolerance scale (Table 4). The heteroplasmy levels in both blood and urine were not correlated with any of the gait parameters (data not shown).

**Table 4:** Correlation analysis (Spearman’s correlation coefficient) of the gait parameters during the normal walking condition and the NMDAS

Variable	NMDAS	NMDAS Subscales			
		*Exercise tolerance*	*Gait stability*	*Myopathy*	*Cerebellar Ataxia*
Velocity (m/s)	**−0** · **472**	*−0* · *399*	**−0** · **599**	*−0* · *505*	*−0* · *450*
Step length (m)	*−0* · *391*	*−0* · *387*	*−0* · *479*	*−0* · *404*	*−0* · *392*
Step time (s)	0 · 165	0 · 098	*0* · *340*	0 · 221	0 · 278
Step width (m)	0 · 206	−0 · 046	0 · 195	−0 · 164	0 · 287
Step length variability (mm)	*0* · *444*	0 · 103	*0* · *489*	−0 · 010	**0** · **601**
Step time variability (ms)	*0* · *505*	0 · 020	**0** · **602**	0 · 120	*0* · *450*
Step width variability (mm)	0 · 246	0 · 192	*0* · *371*	0 · 216	**0** · **547**
Step Length Asymmetry (mm)	−0 · 101	*0* · *337*	0 · 073	−0 · 038	0 · 213
Step Time Asymmetry (ms)	0 · 137	0 · 134	0 · 108	0 · 137	0 · 015

*NMDAS* Newcastle Mitochondrial Disease Adult Scale, *NMDAS* subscales are scored from 0 (no impairments) to 5 (severely impaired), significant correlations with a *p*-value below 0 · 05 are shown in *italic*,

*p*-values below 0 · 001 are **bold**

### NMDAS Subscale analysis

The patient group was subdivided in two smaller groups based on their score on the Gait stability outcome in the NMDAS questionnaire. The gait stability is based on the patients walking performance irrespective of contributing factors such as for example ataxia or muscle weakness. Patients were scored from 0 to 5 where 0 is normal gait stability and 5 is unable to walk without support or falls when standing. In our patient group 8 people had normal gait stability (score 0) and 13 had near normal gait stability (score 1; occasionally difficulties in turns). For this sub analysis both groups were analysed as high gait stability. The remaining patients scored either a 2 (occasionally of balance; *n* = 11) or a 3 (always off balance, occasionally falls; *n* = 3). One patient had no score and was therefore excluded from this analysis. Patients who scored a 2 or higher were analysed as low gait stability. The result of this comparison demonstrates that the high gait stability group had a significantly higher walking step velocity and step length whilst having no significant difference in and step time. Furthermore, the high gait stability group had a lower step length variability, step time variability and, step width variability (Table 5). Overall patients which were classified in the low gait stability group also had higher scores in the subscales myopathy, exercise tolerance and cerebellar ataxia. Whereas the majority of the high stability group scored zero points for these three subscales.

**Table 5:** Subgroup analysis of gait stability in the normal walking condition

Variable	High gait stability (*n* = 21)	Low gait stability (*n* = 14)	Difference between Low and High Gait Stability (*p*-value)
Velocity (m/s)	1 · 19 ± 0 · 15	1 · 02 ± 0 · 18	**0** · **007**
Step length (m)	0 · 62 ± 0 · 06	0 · 56 ± 0 · 08	**0** · **048**
Step time (s)	0 · 53 ± 0 · 04	0 · 55 ± 0 · 07	0 · 077
Step width (m)	0 · 10 ± 0 · 02	0 · 11 ± 0 · 05	0 · 934
Step length variability (mm)	25 · 4 ± 11 · 8	35 · 0 ± 16 · 7	**0** · **003**
Step time variability (ms)	19 · 5 ± 6 · 7	41 · 8 ± 50 · 7	**0** · **001**
Step width variability (mm)	22 · 5 ± 4 · 7	29 · 3 ± 9 · 2	**0** · **022**
Step Length Asymmetry (mm)	13 · 5 ± 10 · 5	21 · 5 ± 18 · 1	0 · 309
Step Time Asymmetry (ms)	7 · 6 ± 7 · 7	10 · 7 ± 10 · 0	0 · 454

High gait stability; a score of 0 or 1 on the topic gait stability of the NMDAS, Low gait stability; a score of 2 or higher on the topic gait stability of the NMDAS, significant

*p*-values below 0 · 05 are shown in **bold** (two-tailed).

## Discussion

To our knowledge this is the first study to investigate the reliability of gait analysis in a group of mitochondrial patients with the m.3243A > G mutation. In accordance with our hypothesis, the results show that all gait parameters can be measured reliably in a group of adult patients with the m.3243A > G mutation and healthy controls using the GAITRite electronic walkway. These findings illustrate that gait measurements are a suitable instrument for intervention studies in patients with the m.3243A > G mutation.

We have tested the gait parameters in four different designs to optimize the protocol for measuring gait in patients with the m.3243A > G mutation. Overall high ICCs were found in all conditions for both patients and control subjects which indicates that the measurements can be reproduced reliably. The highest ICCs for the patient group were found in the post exercise condition and in the after rest condition for the control group. These findings are not in accordance with our initial expectation; we hypothesized that the normal walking condition would have the highest ICCs. The high ICCs during the post exercise condition in the patient group might be a result of the 3MWT in which participants had to walk as fast as possible, which might have led to a steadier pace. After the 3MWT participants got one-minute rest after which they had to walk over the mat. Our results show that people walk faster during the post exercise condition and have a less variable gait pattern, which possibly resulted in higher ICCs. The lowest ICCs were found during the dual task condition in both the patient and control group. The lower ICCs during the dual task condition could be due to the distraction of the dual task while walking [16].

Secondarily we aimed to investigate the differences in gait between patients with the m.3243A > G mutation and healthy controls. In accordance with our hypothesis the results show that patients have a different gait pattern compared to healthy controls. Patients harbouring the m.3243A > G mutation tend to walk significantly slower in all conditions compared to healthy controls, which is possible due to taking smaller steps. Additionally, in three conditions the patients also had a significant higher step width variability whilst no significant difference in mean step width. Galna et al. previously studied a group of eighteen patients with the m.3243A > G mutation and compared their gait characteristics during normal walking to healthy controls. In accordance with their results we also found a reduced step velocity, step length and increased step width variability and step time variability [7]. The similarity in our results with the study of Galna et al. provide further proof for the reliability of this test in measuring similar abnormalities and is therefore suitable for intervention studies. Furthermore, this finding might limit the importance of the age difference between the groups in our study. The higher step width variability in the patient group might indicate towards a reduced postural stability during walking. Interestingly, we did not find a difference in step width variability in the post exercise condition. It is possible that the lower step width variability is linked to an increase in step velocity similar to the findings of temporal gait characteristics reported by Schniepp et al. and Wuehr et al. in patients with cerebral ataxia [17, 18].

Results from the correlation analysis show that patients with more severe clinical symptoms (i.e. a higher NMDAS score) most likely walk slower and make shorter steps. Additionally, these patients have higher step length variability and step time variability. However, the NMDAS questionnaire consists of many subscales that are not all individually linked with gait. The correlations between the total NMDAS questionnaire should therefore be interpreted with caution. Correlations of the four subscales (exercise tolerance, gait stability, myopathy and, cerebral ataxia) were carried out to provide more insight in the correlation between gait specific disease symptoms and the measured gait characteristics. These results indicate that high scores on the subscales gait stability and cerebral ataxia are associated with a lower gait performance. A high gait stability score (i.e. instable gait) is associated with decreased step velocity and step length, an increased step time and gait variability. Additionally, the cerebellar ataxia scale is also highly correlated with gait variability, step velocity and, step length. It is possible that these two scales interfere with each other as people with ataxia often also have balance complaints [16]. Previous studies in elderly at risk of falling also reported that low gait velocities and high gait variability increases the risk of falling (i.e. instable gait) [19, 20]. Our results confirm that lower gait velocities and an increase in gait variability is associated with an increase in gait instability.

In order to look further into the specificity of the NMDAS gait stability subscale we divided the patient group in two subgroups based on the results of their scores. Patients scoring a zero or one on the subscale were marked as high gait stability and patients with a score of two or above were marked as low gait stability. The high stability group walked significantly faster and had a lower gait variability compared to the low stability group. These results suggest that it is possible to differentiate between patients with a good or bad gait stability based on the subscales of the NMDAS. Patients within the low gait stability group also scored worse on the other subscales which might affect gait performance such as myopathy, cerebellar ataxia and exercise tolerance. This finding suggests that the severity of decline in gait is closely related to the manifestation of the different clinical symptoms.

Although we found high ICCs in all conditions this study had some limitations in the dual task and post exercise conditions. The dual task condition caused both controls and patients to be distracted from walking and in some cases participants nearly stopped during walking to perform the given additional task. In those cases, participants had to redo the whole task. This might have contributed to a higher variation in this condition. Our initial goal for the post exercise condition was to examine the effects of exhaustion on the gait pattern in in patients with the m.3243A > G mutation. Although the 3MWT was intensive for most participants they were able to recover within the one-minute resting period. It also seems that all participants adapted the pace they had during the 3MWT which resulted in higher step velocities and possible less variability. Future studies might therefore need to incorporate a different task (e.g. 30 s sit to stand task) to cause exhaustion in participants. However, one should always consider the exhaustion which certain tasks might induce. Therefore, it is important to standardize task rotation to minimize carryover effects of exhausting tasks. A final limitation of this research is the age mismatch between the study groups. The mean age of the control group is lower compared to the patient group. Although the age range is comparable (21–71 years in the control group and 31–68 years in the patient group) the difference in age might have had an influence on the study results. Moreover, in our sample we could not find a significant correlation between age and any gait parameter in the control group nor the patient group. Snijders et al. concluded in their review that gait disorders are not a mere result of aging but often associated with diseases common in elderly people [21]. Based on the results of the NMDAS we indeed observe correlations between clinical symptoms and the different gait parameters. Furthermore, participants in both groups were aged below 60 years of age on average which might have accounted for the low correlations. About 85% of healthy people have close to normal gait at the age of 60 [21].

## Conclusion

In conclusion, we have shown that gait can be measured successful and reliable applying various designs in a group of patients with the m.3243A > G mutation. The recommended method for the assessment of gait is the normal walking method because it resembles daily life activity the most. Walking after exercise might also provide useful information on performance after tiredness however we were unsuccessful in reaching a certain state of exhaustion in our participants. The three-minute walking test in our study did influence the gait parameters but not the reliability in our study. We suggest that the GAITRite is a useful outcome measure for mitochondrial patients and can be a valuable tool for future intervention studies in patients with the m.3243A > G mutation.

## Abbreviations

3MWT: 3 min walking test
ASIS: Anterior superior iliac spine
BBS: Berg balance scale
ICC: Intraclass correlation coefficients
NMDAS: Newcastle mitochondrial disease adults scale
RCMM: Radboud center for mitochondrial medicine
SF-36: Short form 36
