# Erratum to: Quantification of gait in mitochondrial m.3243A > G patients: a validation study

**DOI:** 10.1186/s13023-017-0688-z

**Published:** 2017-10-27

**Authors:** Rob Ramakers, Saskia Koene, Jan T. Groothuis, Paul de Laat, Mirian C. H. Janssen, Jan Smeitink

**Affiliations:** 1 Radboud Center for Mitochondrial Medicine (RCMM) at the Department of Pediatrics, Radboud University Medical Center Nijmegen, Geert Grooteplein Zuid 10, PO BOX 9101, 6500 HB Nijmegen, The Netherlands; 2 Radboud Center for Mitochondrial Medicine (RCMM) at the Department of Internal Medicine, Radboud University Medical Center Nijmegen, Geert Grooteplein Zuid 10, PO BOX 9101, 6500 HB Nijmegen, The Netherlands; 3 Department of Rehabilitation, Donders Institute for Brain, Cognition and Behaviour, Radboud University Medical Center, PO BOX 9101, 6500 HB Nijmegen, The Netherlands

## Erratum

Upon publication of this article [1] it was noticed that Table 2 was incorrect. During Production, errors were introduced into the Variable column. The correct version of Table 2 can be seen below.

**Table 2:** Intra class correlations coefficients (ICCs) of the gait parameters for each walking condition

	Variable	Normal walking	Dual task	Post exercise	After rest
Patients	Velocity (cm/s)	*0·89*	*0·88*	**0·92**	**0·92**
	Step length L (cm)	**0·92**	*0·83*	**0·94**	**0·91**
	Step length R (cm)	**0·90**	*0·87*	**0·94**	**0·93**
	Step time L (s)	0·64	0·72	**0·91**	**0·90**
	Step time R (s)	*0·88*	0·67	**0·90**	**0·91**
	Step width L (cm)	0·77	*0·86*	*0·83*	0·74
	Step width R (cm)	*0·80*	*0·85*	*0·84*	0·76
Controls	Velocity (cm/s)	*0·87*	**0·92**	*0·89*	**0·94**
	Step length L (cm)	**0·90**	*0·88*	**0·92**	**0·93**
	Step length R (cm)	**0·90**	*0·89*	**0·90**	**0·93**
	Step time L (s)	**0·90**	*0·85*	*0·89*	**0·94**
	Step time R (s)	*0·89*	*0·89*	**0·90**	**0·94**
	Step width L (cm)	0·79	*0·80*	0·70	0·76
	Step width R (cm)	*0·80*	0·78	0·70	0·74

*R* right foot, *L* Left foot, ICCs in *italic* are good (above 0·80) and ICCs in bold are perfect (above 0·90), all ICCs were significant with a *p*-value <0·001
